# Hand and shoulder diagnoses are associated with substantial bodily pain compared to vascular complications and long-term glycaemic control in people with long-term type 1 diabetes.

**DOI:** 10.1186/s41687-026-01199-7

**Published:** 2026-09-30

**Authors:** Anne Karin Molvær, Iversen Marjolein Memelink, Jannicke Igland, Mark Peyrot, Grethe S. Tell, Tore Julsrud Berg

**Affiliations:** 1 Centre on Patient-Reported Outcomes, Haukeland University Hospital, Bergen, Norway; 2 Department of Global Public Health and Primary Care, University of Bergen, Bergen, Norway; 3 Department of Health and Caring Sciences, Western Norway University of Applied Sciences, Bergen, Norway; 4 Department of Sociology, Loyola University Maryland, Baltimore, USA; 5 Institute of Clinical Medicine, University of Oslo, Oslo, Norway; 6 Department of Endocrinology, Oslo University Hospital, Oslo, Norway

**Keywords:** Pain, Fatigue, Depression, Quality of life, Frozen shoulder, Trigger finger disorder, Carpal tunnel syndrome, Dupuytren’s disease, Glycemic control, Diabetic vascular complications, Diabetes mellitus, Type 1, Long-term

## Abstract

**Background:**

People with type 1 diabetes are burdened by musculoskeletal hand and shoulder diagnoses and vascular late complications. We aimed to explore associations of musculoskeletal diagnoses, 30-year HbA1c and vascular complications with pain, fatigue, depression, and quality of life (QoL) in an increasing group of long-term survivors of type 1 diabetes.

**Methodology:**

This exploratory, observational study included 104 participants with type 1 diabetes ≥ 45 years’ duration who completed questionnaires assessing pain (RAND-36 bodily pain), fatigue (Fatigue Questionnaire), depression (Patient Health Questionnaire), overall QoL (WHOQoL–BREF) and diabetes-specific QoL (Audit of Diabetes-Dependent QoL). We used linear regression to estimate associations between hand and shoulder diagnoses, 30-year HbA1c ≥ 64 mmol/mol (8%) and vascular complications with PROs.

**Results:**

Compared to 30-year HbA1c and vascular complications, combined hand and shoulder diagnoses showed the strongest estimated association with bodily pain; [B (95% confidence interval)] [-17.4 (-32.8, -2.1)], followed by 30-year HbA1c ≥ 64 mmol/mol [10.9 (-21.9, 0.1)], microvascular complications alone [-11.3 (-24.8, 2.1)], and micro- and macrovascular complications combined [-5.9 (-21.9, 10.2)]. Vascular complications, particularly microvascular, were associated with worse overall QoL and diabetes-specific QoL.

**Conclusions:**

Musculoskeletal hand and shoulder diagnoses show stronger associations with bodily pain than vascular complications, while vascular complications appear more important for QoL in this cohort of long-term survivors with type 1 diabetes. Estimates where however uncertain due to limited sample size.

## Background

People with type 1 diabetes of long duration report more bodily pain, fatigue, depressive symptoms and worse quality of life (QoL) compared to people without diabetes [1]. In addition, they often suffer from musculoskeletal and vascular late complications of diabetes [2–7]. However, the impact of these long-term complications on patient reported outcomes (PROs) is poorly understood.

The prevalence of one or more specific musculoskeletal diagnoses such as trigger finger, carpal tunnel syndrome, Dupuytren’s disease, and frozen shoulder in people with type 1 diabetes has been shown to be as high as 66% after 32 years’ diabetes duration [6] and 94% after more than 45 years’ duration [4]. These diagnoses are generally 2–3 times more common in diabetes compared with the background population [5]. They can be painful and have substantial impact on daily life in people with long-term type 1 diabetes [6, 8–11]. Shoulder pain and stiffness have been associated with total bodily pain in people with long-term type 1 diabetes [12]. However, no studies have investigated whether musculoskeletal hand and shoulder diagnoses are associated with key PRO concepts including bodily pain, fatigue, depression, overall and diabetes-specific QoL in people with long-term type 1 diabetes.

Chronic hyperglycemia is a risk factor for vascular complications in people with diabetes [3, 13] and has been associated with more musculoskeletal hand and shoulder disorders in people with type 1 diabetes [6, 10, 13]. In addition, we have recently shown higher levels of 30-year mean time-weighted HbA1c to be associated with more bodily pain and fatigue in people with long-term type 1 diabetes [1].

Cardiovascular disease and neuropathy may cause pain and are common in people with long-term type 1 diabetes [7]. Many diabetes vascular complications have been associated with severe fatigue in people with longstanding type 1 diabetes [14], however the evidence is inconsistent [15]. Neuropathy has been associated with depressive symptoms in people with long-term type 1 diabetes [2, 16]. Vascular complications and self-reported chest pain have been associated with lower levels of health-related QoL in people with longstanding type 1 diabetes [17, 18].

However, the relative patterns and magnitude of associations of musculoskeletal diagnoses, long-term glycaemic burden and vascular complications with self-reported functioning and well-being remain unclear. This represents a critical knowledge gap, as understanding the relative contribution of these factors is essential for personalized clinical decision making in an increasingly growing group of long-term survivors of diabetes.

Our primary aim was to explore associations of hand and shoulder diagnoses, 30-year HbA1c and vascular complications with pain, fatigue, depression and quality of life in people with long-term type 1 diabetes. As a secondary aim, we subsequently explored associations of specific musculoskeletal diagnoses (trigger finger, carpal tunnel syndrome, Dupuytren’s disease, limited joint mobility of the hands, and frozen shoulder) with relevant PROs in persons with long-term type 1 diabetes.

## Methods

This exploratory work is part of the Dialong study conducted in 2015. All 136 persons diagnosed with type 1 diabetes before 1970 attending the Norwegian Diabetes Centre (NDC) in Oslo, Norway received an invitation by post in 2014.

In total, 104 people (participation rate 76%) attended this study all of Norwegian ethnicity. At the NDC, the study groups’ medical charts were reviewed and data on complications, comorbidities and medications were registered. On their first visit to the NDC, participants answered questionnaires and underwent clinical examinations and interviews. Within three weeks, the participants attended Oslo University Hospital for fasting blood (HbA1c, eGFR) and urine tests (albuminuria), joint examinations, and retinal photos. Detailed data collection methods have been published elsewhere [4, 8]. Sex was defined based on each participant’s preference. The present HbA1c of these patients were like the mean HbA1c of people with type 1 diabetes duration > 45 years in the Norwegian Diabetes Registry for Adults in 2014.

### Patient reported measures

We used Norwegian translated versions of the PRO measures and scored them according to published algorithms [19–23].

### Pain

Total bodily pain was assessed by the bodily pain domain of the general QoL questionnaire RAND-36, which includes two items: ‘How much bodily pain have you had during the past 4 weeks?’, and ‘During the past 4 weeks, how much did pain interfere with your normal work (including both work outside the home and housework)?’, with higher scores indicating better QoL and lower levels of bodily pain (range 0-100) [22].

### Fatigue

Levels of fatigue were measured by the Fatigue Questionnaire (FQ) consisting of 11 items ranging from 0 (better than usual) to 3 (much worse than usual); higher scores represent higher levels of fatigue (range 0–33) [20, 24].

### Depression

Depressive symptoms were assessed by the Patient Health Questionnaire (PHQ-9) [21] covering depressive symptoms experienced during the preceding two weeks. The questionnaire consists of nine items ranging from 0 (not at all) to 3 (nearly every day); higher scores represent more depressive symptoms (range 0–27).

### Quality of Life

Overall QoL was measured by the first overall item of the World Health Organization Quality of Life – BREF (WHOQOL-BREF): During the previous two weeks: ‘How would you rate your quality of life?’ [23], ranging from 1 (very poor) to 5 (very good). Diabetes-specific QoL was measured by the Audit of Diabetes-Dependent Quality of Life (ADDQoL-18), assessing how diabetes affects QoL [19]. Participants rated how their diabetes (negative to positive, range − 3 to + 3) affected the 18 diabetes-specific domains and rated the importance (range + 3 to 0) of each domain. By multiplying the latter two scores for each domain, a weighted score was generated. The average weighted impact scores for each domain were then added up, and the total was divided by the number of applicable domains, generating an average weighted impact score (range − 9 to + 9). For both instruments, higher scores indicate better QoL.

### Hand and shoulder diagnoses

Point prevalence of musculoskeletal diagnoses were established after standard clinical examinations [4] performed by an experienced physician specialized in physical medicine and rehabilitation. The diagnostic criteria were: For trigger finger, a locking sensation of at least one finger present in flexion and ability to extend it by a palpable click [25]; for carpal tunnel syndrome, typical symptoms and a minimum one positive neurological test [26]; for Dupuytren’s disease, one or more fingers were contracted in flexion and had a palpable string of palmar fibromatosis in the same array [27]; for limited joint mobility of the hands, the prayer test [28]; and for frozen shoulder, a reduced passive glenohumeral range of motion of minimum 20° in two planes and normal X-rays of the shoulders [10]. Lifetime prevalence for the same diagnoses was based on a combination of information from chart reviews, clinical examinations, interviews and questionnaires.

### Metabolic parameters

HbA1c was analyzed in fasting morning blood samples. Information on HbA_1_/HbA1c (displayed in mmol/mol and %) available in medical records from the early 1980s up to 2015 were recorded. A mean time-weighted value was calculated from these measurements from the past 14–34 years by calculating the mean HbA1c for each year, then the mean of these values. HbA1c, the transformation of HbA_1_ to HbA1c, and the calculation of mean time-weighted HbA1c have been described previously [4, 29]. Waist circumference was measured in cm at the narrowest part of the waist above the hip bone.

### Vascular complications

Retinopathy, neuropathy, persistent albuminuria and cardiovascular disease have been defined previously [4]. In short, we categorized retinopathy as none or proliferative. We defined neuropathy based on the presence of symmetrical signs in both lower extremities using standard vibration and monofilament tests and on typical symptoms in the lower extremities. We defined persistent albuminuria as a urinary albumin/creatinine ratio of > 2.9 mg/mmol based on two successive samples. For cardiovascular disease, we included total obstructive CAD (previous coronary heart disease including angina pectoris, previous episode of acute coronary syndrome, previous revascularization procedure, obstructive coronary artery disease on computed tomography coronary angiography (CTCA)) [29], a diagnosis of TIA/stroke, and peripheral vascular disease (intermittent claudication, peripheral angiopathy, peripheral bypass).

### Hand and shoulder diagnoses and vascular complications

For hand and shoulder diagnoses we created a four-category variable consisting of: 0 – no problems with hands or shoulders, 1 – hand diagnoses alone defined as at least one diagnosis related to hands, not shoulders, 2 – frozen shoulder alone defined as at least one diagnosis related to shoulders, not hands, 3 – hand and shoulder diagnoses combined, defined as a composite of at least one diagnosis related to both hands and shoulders. For the vascular complications we created a three-category variable consisting of: 0 – no complications, 1 – microvascular complications alone, defined as at least one microvascular complication, not macrovascular, 2 – micro- and macrovascular complications combined, defined as a composite of both micro- and macrovascular complications or macrovascular complications, not microvascular. Since there was only one participant with macrovascular complications and no microvascular complications, it was not possible to include a separate category for participants with macrovascular complications only.

### Statistics

Data are presented as mean ± standard deviation, median (quartiles) or n (%).

We used linear regression to estimate associations between our three main predictor variables (point prevalence of hand and shoulder diagnoses, 30-year mean time-weighted HbA1c ≥ 64 mmol/mol (8%) and vascular complications) and PROs using two different models. The 30-year mean time-weighted HbA1c was dichotomized at 64 mmol/mol (8%) to provide a clinically interpretable measure of long-term glycaemic burden and to facilitate comparison of the strength of the estimated regression coefficient with the categorical musculoskeletal and vascular complication variables. We acknowledge that dichotomization results in some loss of information compared with modelling HbA1c continuously. In the partially adjusted models, we estimated separate regression models for each main predictor, adjusted for age, sex and education. In the fully adjusted models, we included all three main predictor variables in the same model, together with age, sex and education to obtain mutually adjusted regression coefficients for the complications. Results are reported as regression coefficients (*B*) with 95% confidence intervals. Missing data were handled using listwise deletion. Assumptions of normal distribution of residuals and homoscedasticity were assured through histogram of residuals and plots of residuals versus fitted values.

To further explore associations of lifetime prevalence of the musculoskeletal diagnoses with the outcome of bodily pain (RAND-36 bodily pain domain score), we used separate linear regression models for each musculoskeletal diagnosis (trigger finger, carpal tunnel syndrome, Dupuytren’s disease, limited joint mobility of the hands, and frozen shoulder). We used lifetime prevalence of these predictors because the point prevalence for some of these diagnoses was too low to run regression analyses. We applied two levels of adjustments for known, possible confounders. First, for age, sex and education and, second, for mean time-weighted HbA1c and waist circumference. A *P*-value < 0.05 was considered statistically significant. All analyses were performed using STATA/MP 16.1.

## Results

**Fig. 1:**
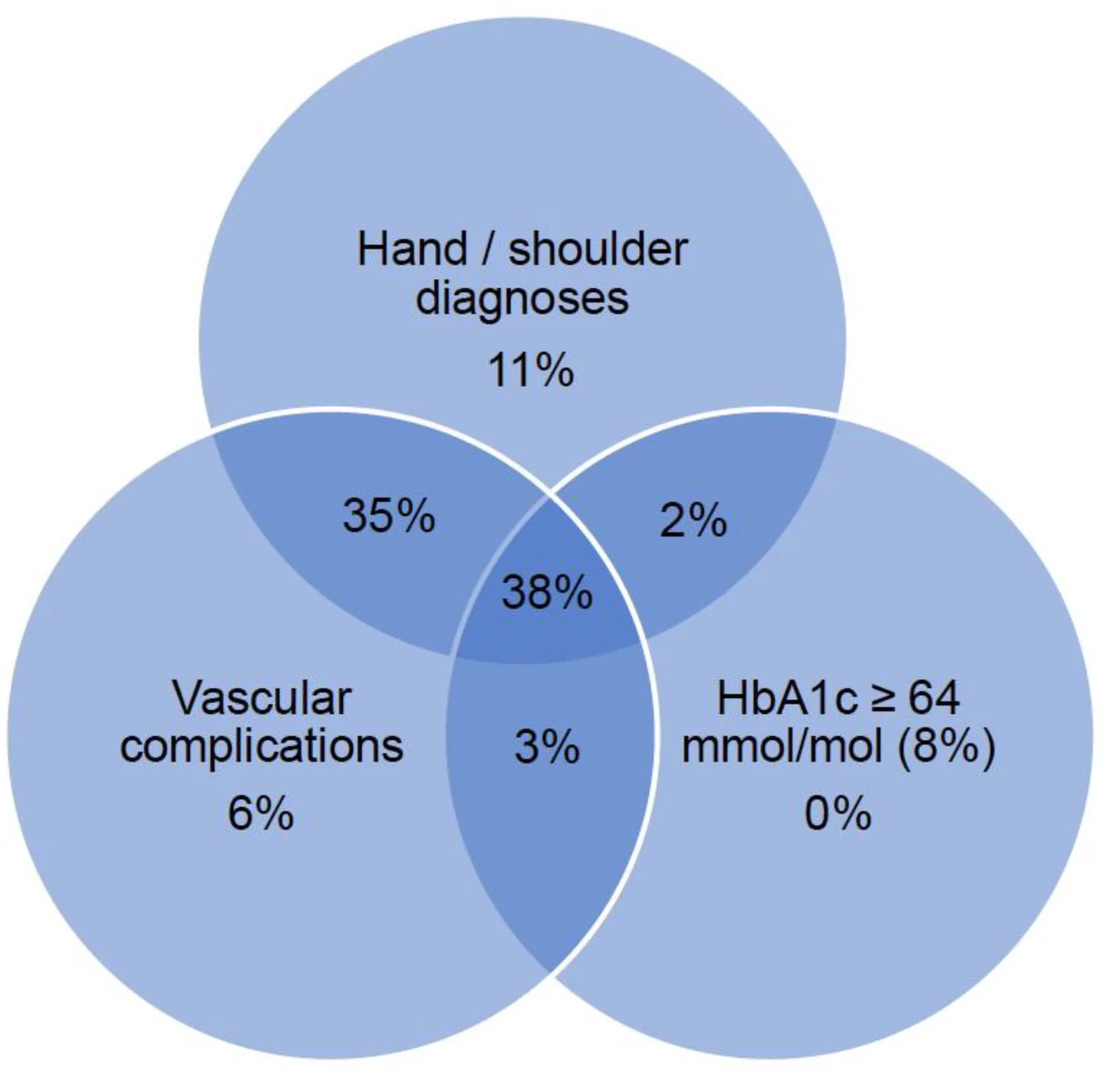
Combinations of hand and shoulder diagnoses, long-term glycemic control and vascular complications. ^a^ Hand and/or shoulder diagnoses consisted of point prevalence of trigger finger, carpal tunnel syndrome or frozen shoulder, and lifetime prevalence of Dupuytren’s disease and limited joint mobility of the hands. ^b^ 30-year HbA1c is mean time-weighted HbA1c the past 14–34 years dichotomized into ≥ 64 mmol/mol (8%). ^c^ Vascular complications consist of microvascular (retinopathy, neuropathy, nephropathy) and cardiovascular disease including total obstructive CAD (previous coronary heart disease including angina pectoris diagnosed by a cardiologist, previous episode of acute coronary syndrome, or previous revascularization procedure; obstructive coronary artery disease on CTCA), a diagnosis of TIA/stroke, and peripheral vascular disease (claudication intermittent (CI), peripheral angiopathy, peripheral bypass)

Participant characteristics (Table 1) include sociodemographic characteristics, questionnaire scores, metabolic parameters, and point and/or lifetime prevalence of hand and shoulder diagnoses and vascular complications. Participants were 61 ± 7.3 years old, equally distributed by sex, 62% were college educated, 25% were living alone, 44% were employed, and median diabetes duration was 49 years. Figure 1 shows the prevalence of hand and shoulder diagnoses, 30-year HbA1c ≥ 64 mmol/mol (8%) and vascular complications, and combinations thereof. In total, 78% of the participants had more than one complication and only 5% had no complications.

**Table 1:** Characteristics of the dialong study population of long-term type 1 diabetes (1; 8; 9)

		*N*
Sociodemographics		
Age, years, mean ± SD	61 ± 7.3	104
Sex male, n (%)	52 (50)	104
Education college, n (%)	64 (62)	104
Living alone, n (%)	26 (25)	104
Employed, n (%)	44 (42)	104
Diabetes specific variable		
Diabetes duration, years	49 (45–67)	104
Questionnaire scores		
RAND-36 Bodily pain domain score [0-100] ^a^, mean ± SD	66 ± 28.2	104
FQ Total fatigue [0–33] ^a^, mean ± SD	15.2 ± 6.1	104
FQ Chronic fatigue (total) (score > 4), n (%)	36 (35)	104
PHQ-9 Depression [0–27] ^a^, mean ± SD	6.3 ± 4.9	104
PHQ-9 Clinically significant depression (score ≥ 10), n (%)	20 (19)	104
WHOQOL-BREF Overall quality of life (QoL) [1–5] ^a^, mean ± SD	3.4 ± 1.1	102
ADDQoL-18 Diabetes-specific QoL [-9 to + 9] ^a^, median (IQR)	-1.3 (-2.3, 0.7)	100
Hand and shoulder diagnoses		
Trigger finger (point prevalence), n (%)	8 (8)	101
Trigger finger (lifetime prevalence), n (%)	42 (42)	101
Carpal tunnel syndrome (point prevalence), n (%)	3 (3)	100
Carpal tunnel syndrome (lifetime prevalence), n (%)	38 (37)	102
Dupuytren’s disease, n (%)	63 (62)	101
Limited joint mobility of the hands, n (%)	51 (50)	102
Frozen shoulder (point prevalence), n (%)	60 (59)	102
Frozen shoulder (lifetime prevalence), n (%)	77 (75)	102
Categories of point prevalence of hand and shoulder diagnoses		
No hand or shoulder diagnoses, n (%)	14 (13)	104
Hand, not shoulder, diagnosis, n (%)	30 (29)	104
Frozen shoulder, no hand diagnosis, n (%)	11 (11)	104
Hand and shoulder diagnoses, n (%)	49 (47)	104
Metabolic parameters		
HbA1c, %, mean ± SD	7.4 ± 0.8	102
HbA1c, mmol/mol, mean ± SD	58 ± 8.6	102
HbA1c – mean time-weighted, %, mean ± SD	7.9 ± 0.8	102
HbA1c – mean time-weighted, mmol/mol, mean ± SD	62.5 ± 8.3	104
Waist circumference, cm, mean ± SD	91.6 ± 12.9	104
Vascular complications		
Retinopathy ^b^, n (%)	45 (43)	104
Neuropathy ^c^, n (%)	66 (64)	104
Persistent albuminuria ^d^, n (%)	17 (16)	104
eGFR < 60	9 (9)	104
Cardiovascular disease ^e^, n (%)	41 (39)	104
Categories of vascular complications		
No vascular complication, n (%)	19 (18)	104
Microvascular, not macrovascular, complications, n (%)	44 (42)	104
Micro- and macrovascular, or macrovascular without microvascular complications, n (%)	41 (39)	104

Abbreviations: FQ – the Fatigue questionnaire; PHQ-9 – the Patient Health Questionnaire; WHOQOL-BREF – the World Health Organization Quality of Life-BREF; ADDQoL-18 – the Audit of Diabetes-Dependent Quality of Life

^a^ Scoring scales. For Patient Health Questionnaire (PHQ-9) and Fatigue Questionnaire (FQ) a higher score indicates more symptoms and is worse. For RAND-36, World Health Organization Quality of Life - BREF (WHOQOL-BREF) and Audit of Diabetes-Dependent Quality of Life (ADDQoL-18) a higher score indicates better quality of life

^b^ Retinopathy was categorized as none or proliferative (pan-retinal photocoagulation scars or proliferative findings) retinopathy based on retina photos

^c^ Neuropathy was defined based on the presence of both typical symptoms in the lower extremities (numbness, unsteadiness, aching or burning pain or pins and needles) and symmetrical signs in both lower extremities using standard monofilament and vibration tests

^d^ Persistent albuminuria was defined as an albumin/creatinine ratio of > 2.9 mg/mmol on two consecutive samples

^e^ Cardiovascular disease included total obstructive CAD (previous coronary heart disease including angina pectoris diagnosed by a cardiologist, previous episode of acute coronary syndrome, or previous revascularization procedure; obstructive coronary artery disease on CTCA) (31), a diagnosis of TIA/stroke, and peripheral vascular disease (intermittent claudication, peripheral angiopathy, peripheral bypass)

### Total bodily pain

In the fully adjusted model, the largest estimated association with bodily pain was observed for combined hand and shoulder diagnoses [B (95% CI) − 17.4 (− 32.8, − 2.1)]. The corresponding estimates were − 13.3 (− 29.8, 3.1) for hand diagnoses alone, − 16.1 (− 36.1, 3.9) for frozen shoulder alone, − 10.9 (− 21.9, 0.1) for 30-year HbA1c ≥ 64 mmol/mol (8%), − 11.3 (− 24.8, 2.1) for microvascular complications alone, and − 5.9 (− 21.9, 10.2) for combined micro- and macrovascular complications. Only combined hand and shoulder diagnoses showed a statistically significant association with bodily pain in the fully adjusted model (Table 2a).

**Table 2:** a, b Linear regression analyses of the associations of musculoskeletal diagnoses, 30-year HbA1c or vascular complications with pain and fatigue in people with long-term type 1 diabetes. The Dialong study (*n* = 104)

T2a	Pain (RAND-36 Bodily pain domain) Scoring scale: 1-100		
	Mean (SD)	Partially adjusted model ^a^ B (95% CI) ^C^	Fully adjusted model ^b^ B (95% CI) ^C^
*Hand and shoulder diagnoses*			
None	81.4 ± 24.6	Ref.	Ref.
Hand diagnoses alone, ≥ one diagnosis	64.3 ± 29.7	-17.9 (-34.2, -1.6)	-13.3 (-29.8, 3.1)
Frozen shoulders alone	68.9 ± 26.3	-17.7 (-38.2, 2.8)	-16.1 (-36.1, 3.9)
Hand and shoulder diagnoses combined, ≥ one diagnosis of each	61.5 ± 27.8	-21.8 (-37.0, -6.6)	-17.4 (-32.8, -2.1)
*Glycemic control*			
30-year HbA1c < 64 *mmol/mol*	72.8 ± 25.3	Ref.	Ref.
30-year HbA1c ≥ 64 mmol/mol	56.6 ± 29.5	-14.3 (-24.4, -4.2)	-10.9 (-21.9, 0.1)
*Vascular complication* ^f^			
None	75.6 ± 23.8	Ref.	Ref.
Microvascular alone, ≥ one diagnosis	62.2 ± 30.98	-17.1 (-30.0, -4.1)	-11.3 (-24.8, 2.1)
Micro- and macrovascular combined, ≥ one diagnosis of each, or macrovascular alone	64.7 ± 25.6	-16.4 (-30.8, -2.1)	-5.9 (-21.9, 10.2)

**T2b**	**Fatigue**,** total (Fatigue Questionnaire)** Scoring scale: 0–33		
	**Mean (SD)**	**Partially adjusted model** ^a^ B (95% CI) ^C^	**Fully adjusted model** ^b^ B (95% CI) ^C^
*Hand and shoulder diagnoses*			
None	12.7 ± 5.3	Ref.	Ref.
Hand diagnoses alone, ≥ one diagnosis	14.7 ± 6.1	2.2 (-1.4, 5.8)	1.1 (-2.6, 4.8)
Frozen shoulders alone	13.5 ± 4.9	1.2 (-3.4, 5.7)	0.8 (-3.7, 5.3)
Hand and shoulder diagnoses combined, ≥ one diagnosis of each	16.7 ± 6.3	4.1 (0.7, 7.4)	2.98 (-0.5, 6.4)
*Glycemic control*			
30-year HbA1c < 64 mmol/mol	14.0 ± 4.95	Ref.	Ref.
30-year HbA1c ≥ 64 mmol/mol	16.8 ± 7.1	1.96 (-0.3, 4.2)	0.8 (-1.7, 3.3)
*Vascular complications*			
None	13.2 ± 5.3	Ref.	Ref.
Microvascular alone, ≥ one diagnosis	15.6 ± 6.4	3.2 (0.4, 6.1)	2.4 (-0.6, 5.4)
Micro- and macrovascular combined, ≥ one diagnosis of each, or macrovascular alone	16.1 ± 5.9	4.3 (1.2, 7,5)	3.1 (-0.5, 6.7)

^a^ Separate model for each of the three types of complications, adjusted for age, sex and education

^b^ All complications mutually adjusted in the same model with additional adjustment for age, sex and education

^C^ Regression coefficients and 95% confidence intervals (CI)

For RAND-36 bodily pain domain: the higher score, the less influence of bodily pain on QoL. For Fatigue Questionnaire: higher score indicates more fatigue

### Fatigue

In the fully adjusted model, hand and shoulder diagnoses combined, microvascular complications alone and micro- and macrovascular complications combined showed similar non-significant associations with fatigue (2.98 (-0.5, 6.4), 2.4 (-0.6, 5.4), 3.1 (-0.5, 6.7)).

### Overall QoL

In the partially adjusted model, hand and shoulder diagnoses combined, microvascular complications alone and micro- and macrovascular combined showed statistically significant associations with worse overall QoL with similar strength. Associations for hand diagnoses alone and frozen shoulder alone had somewhat weaker strength, however not significant. In the fully adjusted model, only microvascular complications alone remained statistically significant (Table 3a).

**Table 3:** a, b Linear regression analyses of the associations of musculoskeletal diagnoses, 30-year HbA1c or vascular complications with QoL in people with long-term type 1 diabetes. The Dialong study (*n* = 104)

T3a	Overall QoL (WHOQOL-BREF) Scoring scale: 1–5		
	Mean (SD)	Partially adjusted model ^a^ B (95% CI) ^C^	Fully adjusted model ^b^ B (95% CI) ^C^
*Hand and shoulder diagnoses*			
None	3.9 ± 1.1	Ref.	Ref.
Hand diagnoses alone, ≥ one diagnosis	3.4 ± 1.2	-0.6 (-1.3, 0.1)	-0.4 (-1.1, 0.3)
Frozen shoulders alone	3.4 ± 0.8	-0.5 (-1.4, 0.3)	-0.5 (-1.3, 0.3)
Hand and shoulder diagnoses combined, ≥ one diagnosis of each	3.3 ± 0.98	-0.7 (-1.3, -0.02)	-0.4 (-1.1, 0.2)
*Glycemic control*			
30-year HbA1c < 64 mmol/mol	3.6 ± 0.99	Ref.	Ref.
30-year HbA1c ≥ 64 mmol/mol	3.2 ± 1.1	-0.4 (-0.8, 0.1)	-0.1 (-0.6, 0.3)
*Vascular complications*			
None	3.9 ± 1.0	Ref.	Ref.
Microvascular alone, ≥ one diagnosis	3.3 ± 1.1	-0.7 (-1.2, -0.1)	-0.6 (-1.1, -0.004)
Micro- and macrovascular combined, ≥ one diagnosis of each, or macrovascular alone	3.2 ± 1.0	-0.8 (-1.4, -0.2)	-0.6 (-1.3, 0.04)

**T3b**	**Diabetes-specific QoL (ADDQoL-18)** Scoring scale: -9 to + 9		
	**Mean (SD)**	**Partially adjusted model** ^a^ B (95% CI) ^C^	**Fully adjusted model** ^b^ B (95% CI) ^C^
*Hand and shoulder diagnoses*			
None	-1.6 ± 1.98	Ref.	Ref.
Hand diagnoses alone, ≥ one diagnosis	-1.7 ± 1.2	-0.1 (-1.1, 0.8)	0.2 (-0.8, 1.2)
Frozen shoulders alone	-1.7 ± 1.1	-0.2 (-1.4, 0.99)	-0.1 (-1.3, 1.0)
Hand and shoulder diagnoses combined, ≥ one diagnosis of each	-1.7 ± 1.5	-0.2 (-1.1, 0.7)	0.04 (-0.9, 0.97)
*Glycemic control*			
30-year HbA1c < 64 mmol/mol	-1.4 ± 1.2	Ref.	Ref.
30-year HbA1c ≥ 64 mmol/mol	-2.0 ± 1.6	-0.6 (-1.2, -0.02)	-0.5 (-1.2, 0.1)
*Vascular complication*			
None	-1.1 ± 0.98	Ref.	Ref.
Microvascular alone, ≥ one diagnosis	-1.99 ± 1.6	-0.98 (-1.7, -0.2)	-0.9 (-1.7, -0.03)
Micro- and macrovascular combined, ≥ one diagnosis of each, or macrovascular alone	-1.6 ± 1.3	-0.7 (-1.5, 0.2)	-0.4 (-1.3, 0.6)

^a^ Separate model for each of the three types of complications, adjusted for age, sex and education

^b^ All complications mutually adjusted in the same model with additional adjustment for age, sex, and education

^C^ Regression coefficients and 95% confidence intervals (CI)

For WHOQOL-BREF and ADDQoL-18: higher score is better

Abbreviations: WHOQOL-BREF – the World Health Organization Quality of Life-BREF; ADDQoL-18 – the Audit of Diabetes-Dependent Quality of Life

### Diabetes-specific QoL

Thirty years HbA1c ≥ 64 mmol/mol (8%) and microvascular complications alone showed statistically significant associations with worse diabetes-specific QoL in the partially adjusted model. In the fully adjusted model, only the coefficient of microvascular complications alone was stable and statistically significant (Table 3b).

*Associations of specific hand and shoulder disorders with total bodily pain*, (Table 4). Among hand and shoulder disorders, lifetime prevalence of trigger finger and frozen shoulder were associated with higher levels of bodily pain after adjustments.

**Table 4:** Linear regression analyses of associations of lifetime prevalence of musculoskeletal diagnoses of the hands and shoulder with total bodily pain in people with long-term type 1 diabetes. The dialong study (*n* = 102)

	Pain (RAND-36 Bodily pain domain)				
	Mean (SD)	Adjusted age, sex, education		Adjusted age, sex, education, mtw HbA1c (mmol/mol), waist circumference	
		B (95% CI) ^a^	*p*-value	B (95% CI) ^a^	*p*-value
No	76.6 ± 24.2				
Trigger finger	51.5 ± 26.1	-18.4 (-29.7, -7.02)	0.002	-16.8 (-27.4, -6.3)	0.002
No	71.6 ± 27.4				
Carpal tunnel syndrome	56.4 ± 26.03	-7.5 (-18.97, 3.9)	0.195	− 7.7 (-18.3, 2.9)	0.151
No	70.4 ± 28.1				
Dupuytren’s disease	63.7 ± 27.6	-10.1 (-20.95, 0.9)	0.070	-3.7 (-14.5, 7.1)	0.503
No	67.9 ± 26.1				
Limited joint mobility of the hands	64.1 ± 29.5	-3.8 (-14.0, 6.5)	0.470	− 4.8 (-14.4, 4.8)	0.319
No	74.5 ± 25.04				
Frozen shoulder	63.2 ± 28.2	-14.9 (-26.4, -3.3)	0.013	-12.1 (-22.99, -1.2)	0.030

For RAND-36 bodily pain domain: the higher score, the less influence of bodily pain on QoL

^a^ Regression coefficient and 95% confidence intervals.Abbreviations: mtw, mean time-weighted

## Discussion

Our main finding in this exploratory study is that, compared to 30-year HbA1c or vascular complications, hand and shoulder diagnoses were more strongly associated with total bodily pain in this unique cohort of long-time survivors of type 1 diabetes. Moreover, hand and shoulder diagnoses and vascular complications showed similarly high associations with fatigue and overall QoL. Finally, microvascular complications alone was the only predictor that remained statistically significantly associated with worse diabetes-specific QoL than hand and shoulder diagnoses, 30-year HbA1c and micro- and macrovascular complications combined. However, the confidence intervals for these estimates overlapped, and no formal statistical comparisons between regression coefficients were performed. Differences in the magnitude of the coefficients should therefore be interpreted descriptively.

The coefficients of hand and shoulder diagnoses were consistent throughout the models and were associated with a clinically relevant difference in pain burden [30] with 16–21% higher levels of pain compared to the reference group in the final model, suggesting an important relationship between these diagnoses with pain, consistent with prior reports [6, 8–11]. (Percent differences between regression coefficients and reference values were calculated as shown in the following example: hand diagnoses alone [-13.3/81.4 = 16%]).

Results from the present and previous studies indicate that trigger finger is common in people with type 1 diabetes [6, 31] also with pain, even after surgical intervention [32], in people with long-term type 1 diabetes. Hand and shoulder diagnoses combined remained statistically significantly associated with pain after adding 30-year HbA1c and vascular complications in the fully adjusted model. However, all the other predictors were associated with a clinically meaningful difference in pain burden, with 15–21% higher levels of pain compared to the reference groups except for micro- and macrovascular complications combined.

Hand and shoulder diagnoses combined, and vascular complications were associated with a clinically relevant difference in fatigue burden, with 18–24% higher levels of fatigue compared to the reference groups. The study is cross-sectional, but these findings could hypothesize that hand and shoulder diagnoses or vascular complications have similar importance on levels of fatigue.

Having hand and shoulder diagnoses or vascular complications showed consistent associations with worse overall QoL through both models. Moreover, in the fully adjusted model all these associations represented 10–15% worse overall QoL compared to the reference groups. These results suggest an important relationship between these diagnoses with QoL regarding vascular complications in line with prior research [17, 18]. Our results emphasize the associations musculoskeletal diagnoses and vascular complications have on overall QoL when both complications are present. However, in diabetes-related QoL, microvascular complications alone remained significantly associated with worse diabetes-specific QoL in the fully adjusted model. This highlights the association between microvascular complications and diabetes-specific QoL, even after adjustment for other long-term complications. Further, our results underline the importance of using both generic and disease-specific PROMs because they are needed to capture the distinct problems experienced by people with chronic conditions. Moreover, a higher recognition of the importance of assessing diabetes-specific QoL alongside biomedical markers is needed to improve the lives of people with diabetes [33].

Hand and shoulder diagnoses, 30-year HbA1c and vascular complications all seem to be associated with PROs in people with long-term type 1 diabetes. This could raise a hypothesis of whether there might exist a yet under-described long-term complication syndrome in people with long duration of type 1 diabetes, consisting of musculoskeletal hand and shoulder disorders, long-term hyperglycaemia and vascular complications affecting PROs such as pain, fatigue and QoL.

The present study has several strengths. The unique population with long-term survivors of type 1 diabetes representing an increasing group of persons with type 1 diabetes, was phenotypically well characterized and had a high response rate on the questionnaires. Furthermore, average HbA1c in our study population was similar to that in the Norwegian Diabetes Registry type 1 diabetes population of more than 45 years’ duration [4]. We used a single value of mean time-weighted HbA1c as a measure of HbA1c in the past, which did not allow us to distinguish between those with different trajectories over time. However, most participants had only minor variation or a slightly decreasing trend in HbA1c over time. Our sample size was relatively small Our sample size was relatively small in relation to the number of parameters included in the fully adjusted regression models, increasing the risk of model overfitting. This may have reduced the precision and stability of the estimates and contributed to the wide confidence intervals and limited statistical power to detect moderate associations. Consequently, non-significant findings should not be interpreted as evidence of absence of an association. Accordingly, he mutually adjusted estimates should be considered exploratory and interpreted with caution.

Residual confounding can also not be excluded, as factors such as physical activity, osteoarthritis, analgesic use, other comorbidities, and measures of adiposity may influence both musculoskeletal diagnoses and PROs. Although waist circumference was included in the analyses of specific musculoskeletal diagnoses, these factors were not comprehensively accounted for in the main models. Given the relatively small sample size, further adjustment would also have increased the risk of model overfitting. Further this cross-sectional study cannot establish causal associations. Thus, interpretation of the findings must be done with caution in this exploratory study. The ethnicity included only people of Norwegian origin. Thus, the results may differ in people of other ethnic origins. The participation rate was 76% which cannot rule out non-response bias. However, the size of the regression coefficients was strong enough to indicate clinically meaningful differences in PROs.

In conclusion, musculoskeletal hand and shoulder diagnoses in people with long-term type 1 diabetes were associated with higher levels of bodily pain. Painful frozen shoulder and trigger finger may be particularly important factors. Combined hand and shoulder diagnoses showed the strongest estimated association for bodily pain among the factors examined. Moreover, hand and shoulder disorders and vascular complications showed associations of similar magnitude with fatigue and overall QoL. This exploratory, cross-sectional study calls for more focus on research to determine whether improved diagnosis and treatment of hand and shoulder disorders may reduce pain and fatigue and improve QoL in people with long-term type 1 diabetes.

## Data Availability

The datasets generated and/or analysed during the current study are not publicly available due to containing confidential clinical trial data, but are available from the corresponding author on reasonable request.
